# D-allulose attenuates dyslipidemia, oxidative stress, and inflammation in high-fat-diet-induced metabolic dysfunction-associated steatotic liver disease in rats

**DOI:** 10.3389/fnut.2026.1982241

**Published:** 2026-10-14

**Authors:** Sami O. Almutairi, Abu ElGasim A. Yagoub, Hassan Barakat

**Affiliations:** 1 Department of Food Science and Nutrition, College of Food and Agricultural Sciences, King Saud University, Riyadh, Saudi Arabia; 2 Department of Food Science and Human Nutrition, College of Agriculture and Food, Qassim University, Buraydah, Saudi Arabia

**Keywords:** anti-inflammatory, antioxidant activity, D-allulose, food supply, lipid metabolism, MASLD, rat model

## Abstract

**Background:**

Metabolic dysfunction-associated steatotic liver disease (MASLD) is a diet-related metabolic disorder characterized by hepatic lipid accumulation, dyslipidemia, insulin resistance, oxidative stress, and chronic inflammation, highlighting the need for safe nutritional interventions.

**Aim:**

This study evaluated the antihyperlipidemic, antioxidant, and anti-inflammatory effects of D-allulose, alone or in combination with dorsomorphin, in a high-fat diet (HFD)-induced rat model of Metabolic dysfunction-associated steatotic liver disease (MASLD).

**Methods:**

Forty-eight male Wistar rats were allocated to a control diet group, an HFD group, or an HFD group treated orally with D-allulose (0.3 or 0.6 g/kg) with or without dorsomorphin (0.2 mg/kg) orally for 12 weeks. Omnibus 𝑝-values for the 22 biochemical endpoints were subjected to a retrospective Benjamini–Hochberg false-discovery-rate sensitivity analysis.

**Results:**

HFD feeding induced weight gain, dyslipidemia, hepatic injury, oxidative stress, inflammation, and hyperinsulinemia. D-allulose dose-dependently ameliorated these disturbances, reducing atherogenic lipids, lipid peroxidation, inflammatory cytokines, and insulin while improving antioxidant status. Notably, TG decreased from 164.51 mg dL^−1^ in HFD rats to 113.52 and 99.43 mg dL^−1^ with low- and high-dose D-allulose, respectively, while LDL-C decreased from 86.45 to 53.19 and 49.24 mg dL^−1^; HDL-C remained unchanged. D-allulose also attenuated HFD-induced hepatic steatosis, degeneration, necrosis, inflammation, and early fibrosis, and this histological protection was maintained or enhanced by dorsomorphin co-administration. A retrospective FDR sensitivity analysis supported the principal lipid, antioxidant, inflammatory, and insulin findings, whereas the ALT and FBG findings did not remain significant and were considered exploratory.

**Conclusion:**

The combination groups generally matched or outperformed D-allulose alone across several biochemical and histological endpoints. However, because dorsomorphin is a non-selective AMPK/BMP inhibitor and AMPK activity was not directly measured, these findings do not establish AMPK’s mechanistic involvement. Overall, D-allulose shows promising protective impacts as a bioactive ingredient for obesity-related metabolic disorders.

## Introduction

Metabolic dysfunction-associated steatotic liver disease (MASLD), formerly known as non-alcoholic fatty liver disease (NAFLD), has emerged as a major public-health concern, impacting 25–30% of adults globally. It is closely linked to the global rise in obesity, metabolic syndrome, and type 2 diabetes mellitus (1–3). It encompasses a spectrum of hepatic disorders characterized by excessive hepatic triglyceride accumulation in the absence of significant alcohol intake. It can progress from simple steatosis to metabolic dysfunction-associated steatohepatitis (MASH), formerly named non-alcoholic steatohepatitis (NASH), fibrosis, and, in advanced cases, cirrhosis or hepatocellular carcinoma (3). The pathophysiology of MASLD is increasingly viewed through a “multiple parallel-hit” framework, in which insulin resistance, adipose tissue dysfunction, mitochondrial disturbances, and dysregulated lipid metabolism interact to promote hepatic fat deposition and subsequent liver injury (4–6).

Central to the progression of MASLD are 3 interlinked pathways, including oxidative stress, lipid dysregulation, and chronic, low-grade inflammation (7). Excessive free-fatty-acid flux into the liver, coupled with impaired mitochondrial β-oxidation and endoplasmic reticulum stress, leads to the accumulation of toxic lipid intermediates and the generation of reactive oxygen species (8). This imbalance between oxidant production and antioxidant defenses triggers lipid peroxidation, hepatocyte damage, and the release of damage-associated molecular patterns that activate Kupffer cells and infiltrating immune cells (9). The ensuing inflammatory cascade, driven by cytokines such as TNF-α, IL-6, and IL-1β, perpetuates hepatocellular injury, promotes fibrogenesis, and accelerates the transition from steatosis to MASH (7).

Given this multifactorial landscape, current therapeutic strategies remain limited and largely supportive (10). Lifestyle interventions centered on weight reduction, physical activity, and dietary modification are considered first-line management, yet adherence is often suboptimal, and outcomes are variable (11). Despite recent progress that has led to the development of pharmacotherapies, such as resmetirom (a thyroid hormone receptor-β agonist licensed for MASH with fibrosis), these drugs target specific pathways, may have adverse effects, and are indicated only for severe illness (12). Therefore, there is a growing interest in functional food ingredients that can safely and simultaneously target various pathomechanisms by addressing oxidative stress, lipid accumulation, and inflammation (13, 14).

In this context, D-allulose, a rare C-3-epimer of fructose, has attracted interest as a low-calorie sugar substitute with favorable metabolic properties (15). Unlike typical sugars, D-allulose is poorly metabolized in humans and contributes minimally to net energy intake. It also influences hepatic glucose and lipid pathways by modulating enzymes and genes involved in gluconeogenesis, fatty acid β-oxidation, cholesterol metabolism, and bile-acid homeostasis (16). Experimental studies in rodent models of diet-induced obesity indicate that D-allulose can attenuate hepatic steatosis, improve dyslipidemia, and enhance insulin sensitivity, suggesting a potential hepatoprotective profile (17). Recent evidence further points to its ability to modulate macrophage function, reduce pro-inflammatory chemokine signaling, and improve mitochondrial energetics, implicating D-allulose in both metabolic and immune-related aspects of liver injury (18).

Despite these promising observations, several crucial gaps remain in the research. First, no research has comprehensively investigated the integrated, multi-target effects of D-allulose on MASLD pathophysiology, particularly its antioxidant, lipid-regulatory, and anti-inflammatory actions in a single well-defined MASLD rat model (16). Most prior work has focused on isolated endpoints or mechanistic pathways (17, 18). Further, data on dosage response for low and high D-allulose doses across all three pathogenic axes (lipogenesis, oxidative stress, and fibrosis) are lacking (19). There is a lack of studies examining the effects of D-allulose with and without AMPK inhibition. Although AMPK signaling has been proposed to mediate the metabolic effects of D-allulose (20), the relative contributions of AMPK versus other pathways (e.g., PPARα and the gut microbiota) remain unknown. In the present study, however, we did not directly quantify hepatic AMPK phosphorylation or downstream targets such as SIRT1, PGC-1α, or Nrf2. Hence, the involvement of AMPK remains hypothetical and cannot be confirmed mechanistically.

Accordingly, the present study addresses these gaps by evaluating the antihyperlipidemic, antioxidant, and anti-inflammatory effects of D-allulose in HFD-fed rats comprehensively; comparing two doses of D-allulose (0.3 and 0.6 g/kg) to identify the dose–response relationships; and utilizing dorsomorphin, a pharmacological AMPK inhibitor, to determine the contribution of AMPK signaling to the protective effects of D-allulose. Therefore, the overarching hypothesis is that dietary supplementation with D-allulose will ameliorate hepatic lipid accumulation, enhance antioxidant defenses, and suppress pro-inflammatory signaling in a dose-dependent manner. That co-administration of dorsomorphin was used to probe the contribution of AMPK signaling to these effects, while recognizing that AMPK involvement remains a working hypothesis. In light of these considerations, this study aims to investigate the effects of low and high doses of D-allulose on body weight (BW), liver and kidney functions, and antioxidant, antihyperlipidemic, and anti-inflammatory biomarkers in an HFD-fed MASLD rat model.

## Materials and methods

### Animals

A total of 48 adult male Wistar albino rats, aged 6–8 weeks and weighing 150–180 g, were obtained from the Animal House Facility at King Saud University, Riyadh, Saudi Arabia. The animals were housed in standard polypropylene cages under controlled environmental conditions of 22 ± 2 °C and 45 ± 5% relative humidity, with a 12-h light–dark cycle. Diet and water were provided *ad libitum* throughout the experimental period to ensure optimal nutritional status and welfare.

### Ethical statements

All experimental procedures, including euthanasia, were conducted in strict accordance with the Guide for the Care and Use of Laboratory Animals published by the National Academies Press (21). The study protocols were additionally approved by the Research Ethics Committee at King Saud University, Riyadh, Saudi Arabia, ensuring full compliance with national and international ethical standards.

### Diets, D-allulose, and dorsomorphin

The rats were randomly assigned to experimental groups and fed either a control diet (CD) or a high-fat diet (HFD) for 12 weeks. The control diet, providing 10% of total calories from fat, 20% from protein, and 70% from carbohydrates, with an energy density of 3.85 to 4.05 kcal/g (Diet code D12450B), and the HFD, providing 60% of total calories from fat 20% from protein, and 20% from carbohydrates, yielding an energy density of 5.21 kcal/g, were formulated according to the Research Diets, Inc. (Diet code D12492; New Brunswick, NJ, USA) guidelines. Detailed macronutrient composition and energy density of both diets are summarized in Supplementary Table S2. D-Allulose (purity >99%) was obtained from Xi’an Aogu Biotech Co., Ltd. (Shaanxi, China), and dorsomorphin, an AMPK inhibitor (purity ≥99.1%), was purchased from MyBioSource (San Diego, CA, USA).

### Experimental design

After a 14-day adaptation period, the rats were randomly allocated into six experimental groups (*n* = 8 per group) as follows: Group 1, a normal diet control group fed a standard chow diet (CD) and supplemented with saline (5 mL); Group 2, an HFD control group fed a high-fat diet (HFD) and supplemented with saline (5 mL); Groups 3 and 4, treatment groups fed HFD and supplemented with oral D-allulose at 0.3 g kg^−1^ (HFDA1) or 0.6 g kg^−1^ BW (HFDA2); and Groups 5 and 6, additional groups fed HFD and supplemented with D-allulose (0.3 g kg^−1^, orally) together with 0.2 mg kg^−1^ BW dorsomorphin (HFDA1 + D) or D-allulose (0.6 g kg^−1^, orally) together with 0.2 mg kg^−1^ BW dorsomorphin (HFDA2 + D). D-allulose was dissolved in sterile distilled water to the desired concentration for oral administration, and dorsomorphin (as the dihydrochloride salt) was dissolved in PBS (pH 7.2) at 0.2 mg kg^−1^ BW for oral administration. Dorsomorphin, a non-selective AMPK/BMP inhibitor with known off-target actions, used here as a pharmacological probe of AMPK-related signaling, was administered to Groups 5 and 6 to assess the contribution of AMPK signaling to the hypoglycemic and hypolipidemic effects of D-allulose. We chose the oral route for dorsomorphin to harmonize administration with D-allulose and minimize repeated injections and handling stress; however, pharmacokinetic data for this regimen are limited, and systemic AMPK inhibition was not directly confirmed. The selected D-allulose and dorsomorphine dose levels were based on a previous experimental study (22) demonstrating favorable metabolic responses at comparable levels in rodent models (23, 24). Throughout the 12-week intervention, all animals were monitored Biweekly for changes in BW and, at the end of the study, underwent comprehensive metabolic assessments; blood and tissue samples were collected for subsequent biochemical, histological, and molecular analyses.

### Blood and tissue collection

At the end of the 12-week experimental period, following a 12-h fasting period, rats were deeply anesthetized (25) by intraperitoneal injection of ketamine hydrochloride (80 mg/kg body weight; Ketamine 10%, Alfasan International B. V., Woerden, The Netherlands) combined with xylazine hydrochloride (10 mg/kg body weight; Xylazine 2%, Alfasan International B. V., Woerden, The Netherlands). Adequate surgical anesthesia was confirmed by the absence of the pedal withdrawal reflex. Blood samples were then collected by terminal cardiac puncture, left to stand at room temperature for 30 min, and then centrifuged to obtain serum. Immediately after blood collection, euthanasia was completed by cervical dislocation. Death was confirmed by the absence of spontaneous respiration and heartbeat before tissue collection. The liver, kidneys, and epididymal adipose tissue were subsequently excised for histological evaluation (26).

### Determination of fasting blood glucose level, lipid profile, liver and kidney functions

Fasting blood glucose (FBG, expressed as mg dL^−1^) was measured by an enzymatic colorimetric assay kit based on the glucose oxidase - phenol + 4-aminoantipyrine (GOD-PAP) method. The lipid profile was assessed using enzymatic colorimetric kits: triglycerides (TG, mg dL^−1^) and total cholesterol (TCHOL, mg dL^−1^) were determined with a GPO-PAP-based assay, while high-density lipoprotein cholesterol (HDL-C, mg dL^−1^) was quantified using a direct homogeneous enzymatic colorimetric kit, following the manufacturer’s instructions. Low-density lipoprotein (LDL-C, mg dL^−1^) and very-low-density lipoprotein (VLDL-C, mg dL^−1^) levels were then estimated using the Friedewald et al. (27). In addition, the HDL-C/TCHOL ratio was calculated as an additional indicator of cardioprotective lipoprotein balance. Serum activities of hepatic enzymes, including alanine aminotransferase (ALT, IU L^−1^), aspartate aminotransferase (AST, IU L^−1^), and alkaline phosphatase (ALP, IU L^−1^), along with total bilirubin (T. Bili, mg dL^−1^) and direct bilirubin (D. Bili, mg dL^−1^), were evaluated using commercially available assay kits. Measurements were performed with an alanine aminotransferase kit (EC 2.6.1.2), an aspartate aminotransferase kit (EC 2.6.1.1), an optimized alkaline phosphatase kit (EC 3.1.3.1), and photometric test kits for total and direct bilirubin, following the manufacturer’s protocols.

Renal function parameters, namely serum total protein (T. protein, g dL^−1^), albumin (g dL^−1^), creatinine (mg dL^−1^), and urea (mg dL^−1^), were quantified using photometric and colorimetric assay kits based on the Biuret method for total protein, the bromocresol green (BCG) method for albumin, urease-glutamate dehydrogenase (GLDH) UV-kinetic method for urea, and a fully enzymatic kit applying the GLDH method for creatinine, as recommended by the supplier. Globulin (g dL^−1^) was calculated as the difference between total protein and albumin. Blood urea nitrogen (BUN, mg dL^−1^) was derived by multiplying the urea concentration by 0.47. All used biochemical assay kits were obtained from Human GmbH (Wiesbaden, Germany). The atherogenic index (AI) was computed based on the formula described by Nwagha et al. (28).

### Serum oxidative stress biomarkers

Reduced glutathione (GSH, expressed as μg dL^−1^) was quantified using a colorimetric GSH assay kit (E-BC-K030-S, Elabscience, USA), following the procedure outlined by Beutler et al. (29). Lipid peroxidation was assessed by measuring malondialdehyde (MDA, nmol mL^−1^) with a thiobarbituric acid reactive substances (TBARS)-based colorimetric kit (E-BC-K025-S, Elabscience, USA), in which MDA forms a chromogenic adduct with thiobarbituric acid (TBA); the concentration was calculated using the method of Ohkawa et al. (30). After completion of the reaction, the absorbance of the supernatant was read at 532 nm and reported in nmol mL^−1^. Superoxide dismutase (SOD, expressed as U L^−1^) activity was determined using an SOD assay kit (E-BC-K022-S, Elabscience, USA), following the protocol described by Giannopolitis and Ries (31). The colorimetric reaction was measured at 550 nm and converted to units per liter. Catalase (CAT, U L^−1^) activity was measured with a CAT activity assay kit (E-BC-K031-S, Elabscience, USA), following the method reported by Aebi (32). All oxidative stress biomarkers were measured using a clinical blood chemistry analyzer (HumaLyzer 4,000, Human, Germany) in accordance with the manufacturer’s technical specifications.

### Pro-inflammatory cytokines TNF-α and IL-6

The TNF-α and IL-6 levels were measured in serum samples using commercially available enzyme-linked immunosorbent assay (ELISA) kits (eBioscience, Thermo Fisher Scientific, USA), according to the manufacturer’s instructions. Briefly, serum was thawed on ice and centrifuged at 1,000 × g for 10 min at 4 °C to remove particulates, then diluted in the provided assay diluent to fall within the kit’s linear range. Pre-coated 96-well microplates were incubated with standards, blanks, and diluted samples for 2–3 h at room temperature to allow binding of TNF-α or IL-6 to the immobilized capture antibody. After washing with wash buffer to remove unbound material, a biotin-conjugated detection antibody specific for TNF-α or IL-6 was added and incubated for a further 1 h, followed by horseradish peroxidase-labeled streptavidin for 30 min. The reaction was developed by adding the tetramethylbenzidine (TMB) substrate solution and stopped after a defined incubation period with the provided stop solution. Optical density was read at 450 nm using a microplate reader, and TNF-α and IL-6 concentrations were calculated from the corresponding standard curve generated in each assay plate, using four- or five-parameter logistic curve-fit software. All assays were performed in duplicate, and intra-assay and inter-assay coefficients of variation were within the manufacturer-specified acceptable limits.

### Histopathological examination

Autopsies of the fixed Liver in 10% formalin-saline for up to 48 h were performed on different experimental groups. For dehydration, samples were washed with water, then diluted in a series of alcohols (methyl, ethyl, and absolute ethyl). At 56 °C, liver tissue was processed using standard paraffin-embedding, microtome sectioning, and hematoxylin–eosin staining, as described by Kim Suvarna and Layton (33). Slides were examined and photomicrographs captured using a Nikon Eclipse E800 microscope (Melville, NY, USA) equipped with an Olympus digital camera. Subsequently, photomicrographs were taken, followed by blind evaluation of architectural changes and lesion severity by two pathologists. Based on the severity of the microscopic lesions, all liver specimens were examined for histological alterations and classified on a modified semi-quantitative scale as none (−), mild (+), moderate (++), or severe (+++). Lesion severity was graded as absent (−), mild (+), moderate (++), or severe (+++), which equal (0 for <5%), (1 for 5–33%), (2 for 33–66%), and (3 for >66%) according to Liang et al. (34).

### Statistical analysis

Statistical analysis was performed using SPSS version 22.0 (IBM Corp., Armonk, NY, USA). Results are presented as mean ± standard error of the mean (SEM), with *n* = 8 animals per group. Normality was assessed using the Shapiro–Wilk test, and homogeneity of variance was evaluated using Levene’s test. Differences among the six experimental groups were assessed separately for each endpoint using one-way analysis of variance, followed by Tukey’s post-hoc test for pairwise comparisons according to Steel et al. (35). Nominal *p*-values below 0.05 were initially considered statistically significant. Because 22 biochemical endpoints were evaluated in Tables 1–5, a retrospective sensitivity analysis was subsequently performed by applying the Benjamini–Hochberg procedure to the reported omnibus ANOVA 𝑝-values to control the false discovery rate at 5%. Both nominal and FDR-adjusted *p*-values are reported. Because the sensitivity analysis used the rounded *p*-values reported in the manuscript, including values of 0.001, the adjusted values should be considered approximate. Findings with FDR-adjusted *p* < 0.055 were considered robust to correction, whereas findings significant only at the nominal level were considered exploratory. Tukey’s test controlled pairwise comparisons within individual endpoints but did not control multiplicity across the complete battery of endpoints.

**Table 1:** Antihyperlipidemic effect of D-allulose alone or in combination with dorsomorphin in HFD-fed rats (mean±SEM), *n* = 8.

Groups	Serum lipid profile (mg dL^−1^)					
	TG	TCHOL	HDL-C	LDL-C	VLDL-C	HDL-C/TCHOL
CD	65.17 ± 1.84^e^	116.03 ± 2.44^b^	39.52 ± 2.49^a^	63.48 ± 1.04^b^	13.03 ± 0.37^e^	0.32 ± 0.01^b^
HFD	164.51 ± 12.89^a^	152.60 ± 4.92^a^	33.25 ± 0.50^b^	86.45 ± 5.89^a^	32.90 ± 2.58^a^	0.22 ± 0.01^c^
HFDA1	113.52 ± 3.69^b^	114.28 ± 3.72^b^	38.38 ± 5.17^a^	53.19 ± 8.15^bc^	22.71 ± 0.74^b^	0.34 ± 0.06^ab^
HFDA2	99.43 ± 2.99^bc^	108.65 ± 4.39^bc^	39.52 ± 2.14^a^	49.24 ± 1.95^c^	19.89 ± 0.60^bc^	0.37 ± 0.01^ab^
HFDA1 + D	88.68 ± 3.12^cd^	102.32 ± 1.22^cd^	38.19 ± 0.87^a^	46.39 ± 0.59^c^	17.74 ± 0.62^cd^	0.37 ± 0.00^ab^
HFDA2 + D	78.91 ± 3.13^de^	96.70 ± 1.27^d^	40.28 ± 0.69^a^	40.63 ± 1.18^c^	15.78 ± 0.63^de^	0.42 ± 0.01^a^
Nominal *P*-value	0.001	0.001	0.460	0.001	0.001	0.005
FDR-adjusted *P*-value	0.002	0.002	0.506	0.002	0.002	0.009

CD, normal control group fed a standard chow diet; HFD, positive control group fed a HFD; HFDA1, HFD rats supplemented with oral D-allulose at 0.3 g kg^−1^ bw; HFDA2, HFD rats supplemented with oral D-allulose at 0.6 g kg^−1^ bw; HFDA1 + D, HFD rats supplemented with D-allulose at 0.3 g kg^−1^ bw orally plus dorsomorphin at 0.2 mg kg^−1^ bw; HFDA2 + D, HFD rats supplemented with D-allulose at 0.6 g kg^−1^ bw orally plus dorsomorphin at 0.2 mg kg^−1^ bw; ^a,b,c,d^, means sharing same letter do not differ significantly at *p* < 0.05 by one way ANOVA followed by Tukey’s test.

**Table 2:** Effect of D-allulose alone or in combination with dorsomorphin on Liver function parameters of HFD-fed rats (mean±SEM), *n* = 8.

Groups	Liver’s function				
	ALT (U L^−1^)	AST (U L^−1^)	T. Protein (g dL^−1^)	Albumin (g dL^−1^)	Globulin (g dL^−1^)
CD	34.50 ± 2.31^b^	57.91 ± 5.91^ab^	7.88 ± 0.49^a^	4.82 ± 0.37^a^	3.07 ± 0.17^a^
HFD	64.69 ± 3.89^a^	69.12 ± 4.01^a^	8.00 ± 0.44^a^	4.88 ± 0.37^a^	3.11 ± 0.07^a^
HFDA1	32.34 ± 6.57^b^	50.13 ± 4.07^b^	6.70 ± 0.22^a^	3.69 ± 0.22^a^	3.00 ± 0.11^a^
HFDA2	33.34 ± 2.88^b^	52.66 ± 3.01^ab^	7.14 ± 0.56^a^	4.14 ± 0.66^a^	3.00 ± 0.15^a^
HFDA1 + D	33.51 ± 4.47^b^	48.13 ± 8.89^b^	7.81 ± 0.58^a^	4.77 ± 0.58^a^	3.03 ± 0.02^a^
HFDA2 + D	34.50 ± 4.54^b^	53.15 ± 4.45^ab^	6.47 ± 0.38^a^	3.52 ± 0.48^a^	2.95 ± 0.11^a^
Nominal *P*-value	0.050	0.001	0.149	0.222	0.931
FDR-adjusted *P*-value	0.073	0.002	0.182	0.257	0.931

CD, normal control group fed a standard chow diet; HFD, positive control group fed an HFD; HFDA1, HFD rats supplemented with oral D-allulose at 0.3 g kg^−1^ bw; HFDA2, HFD rats supplemented with oral D-allulose at 0.6 g kg^−1^ bw; HFDA1 + D, HFD rats supplemented with D-allulose at 0.3 g kg^−1^ bw orally plus dorsomorphin at 0.2 mg kg^−1^ bw; HFDA2 + D, HFD rats supplemented with D-allulose at 0.6 g kg^−1^ bw orally plus dorsomorphin at 0.2 mg kg^−1^ bw. ^a,b^, sharing the same letter do not differ significantly at *p* < 0.05 by one-way ANOVA followed by Tukey’s test. One unit (1 U) of ALT activity is defined as the amount of enzyme that catalyzes the formation of 1.0 μmol of pyruvate (or an equivalent reaction product) per minute at a standard temperature of 37 °C (93). One unit (1 U) of Aspartate Aminotransferase (AST) activity is defined as the amount of the enzyme that catalyzes the conversion of 1.0 μmol of substrate (such as aspartate and α-ketoglutarate) to reaction products (such as glutamate and oxaloacetate) per minute under standardized, specific assay conditions (94).

**Table 3:** Effect of D-allulose alone or in combination with dorsomorphin on the kidneys’ functions of HFD-fed rats (mean ± SEM), *n* = 8.

Group	Kidneys’ functions		
	Creatinine (mg dL^−1^)	Urea (mg dL^−1^)	BUN (mg dL^−1^)
CD	0.76 ± 0.05^a^	45.95 ± 0.98^ab^	21.46 ± 0.46^ab^
HFD	0.91 ± 0.09^a^	54.62 ± 0.81^a^	25.51 ± 0.38^a^
HFDA1	0.87 ± 0.10^a^	50.90 ± 5.56^ab^	23.77 ± 2.60^ab^
HFDA2	0.78 ± 0.09^a^	42.68 ± 4.65^b^	19.93 ± 2.17^b^
HFDA1 + D	0.75 ± 0.01^a^	45.83 ± 0.63^ab^	21.40 ± 0.29^ab^
HFDA2 + D	0.77 ± 0.09^a^	44.37 ± 1.66^ab^	20.72 ± 0.77^ab^
Nominal *P*-value	0.637	0.131	0.136
FDR-adjusted *P*-value	0.667	0.176	0.176

CD, normal control group fed a standard chow diet; HFD, positive control group fed an HFD; HFDA1, HFD rats supplemented with oral D-allulose at 0.3 g kg^−1^ bw; HFDA2, HFD rats supplemented with oral D-allulose at 0.6 g kg^−1^ bw; HFDA1 + D, HFD rats supplemented with D-allulose at 0.3 g kg^−1^ bw orally plus dorsomorphin at 0.2 mg kg^−1^ bw; HFDA2 + D, HFD rats supplemented with D-allulose at 0.6 g kg^−1^ bw orally plus dorsomorphin at 0.2 mg kg^−1^ bw; ^a,b,c,d^, means sharing the same letter does not differ significantly at *p* < 0.05 by one-way ANOVA followed by Tukey’s test.

**Table 4:** Effect of D-allulose alone or in combination with dorsomorphin on antioxidant biomarkers in HFD-fed rats (mean ± SEM), *n* = 8.

Groups	Antioxidant biomarkers			
	GSH (μg dL^−1^)	MDA (nmol mL^−1^)	CAT (U L^−1^)	SOD (U L^−1^)
CD	56.40 ± 7.89^a^	19.41 ± 0.55^d^	81.60 ± 7.50^a^	82.62 ± 8.30^a^
HFD	22.32 ± 4.06^b^	33.20 ± 0.48^a^	53.71 ± 13.33^b^	44.87 ± 3.78^c^
HFDA1	34.49 ± 4.42^b^	23.24 ± 0.69^b^	57.45 ± 9.29^b^	55.96 ± 4.80^bc^
HFDA2	52.04 ± 8.67^a^	22.23 ± 0.68^bc^	64.73 ± 4.79^a^	58.41 ± 6.12^bc^
HFDA1 + D	51.10 ± 1.25^a^	19.92 ± 0.39^d^	77.22 ± 5.23^a^	71.04 ± 6.84^ab^
HFDA2 + D	51.99 ± 2.64^a^	20.76 ± 0.32^cd^	74.56 ± 10.10^a^	77.83 ± 5.72^a^
Nominal *P*-value	0.001	0.001	0.017	0.001
FDR-adjusted *P*-value	0.002	0.002	0.029	0.002

CD, normal control group fed a standard chow diet; HFD, positive control group fed an HFD; HFDA1, HFD rats supplemented with oral D-allulose at 0.3 g kg^−1^ bw; HFDA2, HFD rats supplemented with oral D-allulose at 0.6 g kg^−1^ bw; HFDA1 + D, HFD rats supplemented with D-allulose at 0.3 g kg^−1^ bw orally plus dorsomorphin at 0.2 mg kg^−1^ bw; HFDA2 + D, HFD rats supplemented with D-allulose at 0.6 g kg^−1^ bw orally plus dorsomorphin at 0.2 mg kg^−1^ bw; ^a,b,c,d^, means sharing the same letter, do not differ significantly at *p* < 0.05 by one-way ANOVA followed by Tukey’s test. One unit (1 U) of SOD activity was defined as the amount of enzyme required to inhibit NBT reduction by 50% (95). One unit (1 U) of CAT activity is universally defined as the amount of enzyme that decomposes 1 μmol of H₂O₂ per minute under standardized assay conditions (typically at pH 7.0 and 25 °C) (96).

**Table 5:** Effect of D-allulose alone or in combination with dorsomorphin on inflammatory and endocrine biomarkers in HFD-fed rats (mean ± SEM), *n* = 8.

Groups	Inflammatory and endocrine biomarkers			
	IL-6 (pg mL^−1^)	TNF-α (pg mL^−1^)	Insulin (ng mL^−1^)	FBG (mg dL^−1^)
CD	9.29 ± 0.07^d^	23.64 ± 0.10^c^	2.24 ± 0.87^c^	96.85 ± 10.16^b^
HFD	17.49 ± 0.53^a^	49.73 ± 2.18^a^	15.88 ± 0.79^a^	129.29 ± 9.97^a^
HFDA1	14.17 ± 0.07^b^	32.99 ± 0.64^b^	10.35 ± 4.30^ab^	101.87 ± 2.72^b^
HFDA2	13.64 ± 0.33^b^	32.80 ± 0.72^b^	7.24 ± 1.18^bc^	85.41 ± 14.24^b^
HFDA1 + D	13.48 ± 0.16^b^	23.38 ± 0.38^c^	5.35 ± 0.34^bc^	100.89 ± 2.07^b^
HFDA2 + D	12.36 ± 0.13^c^	23.45 ± 0.23^c^	3.35 ± 0.97^c^	93.29 ± 3.84^b^
Nominal *P*-value	0.001	0.001	0.003	0.047
FDR-adjusted *P*-value	0.002	0.002	0.006	0.073

CD, normal control group fed a standard chow diet; HFD, positive control group fed an HFD; HFDA1, HFD rats supplemented with oral D-allulose at 0.3 g kg^−1^ bw; HFDA2, HFD rats supplemented with oral D-allulose at 0.6 g kg^−1^ bw; HFDA1 + D, HFD rats supplemented with D-allulose at 0.3 g kg^−1^ bw orally plus dorsomorphin at 0.2 mg kg^−1^ bw; HFDA2 + D, HFD rats supplemented with D-allulose at 0.6 g kg^−1^ bw orally plus dorsomorphin at 0.2 mg kg^−1^ bw. ^a,b,c,d^, means sharing the same letter do not differ significantly at *p* < 0.05 by one-way ANOVA followed by Tukey’s test.

## Results

### The effect of treatments on weight gain percentage

Figure 1 illustrates that the weight-gain curve shows a clear HFD-induced increase over 12 weeks, with the HFD group rising fastest and reaching the highest final gain. In contrast, all D-allulose-treated groups showed a lower trajectory, especially at the higher dose and in the presence of dorsomorphin. The weight-gain percentages were 51.26, 60.37, 57.46, 53.73, 43.82, and 42.12 for CD, HFD, HFDA1, HFDA2, HFDA1 + D, and HFDA2 + D, respectively. The presented profile indicates substantial suppression of HFD-driven weight gain, as indicated by a significant difference (*p* < 0.05). The pattern suggests a dose-related benefit, with the higher D-allulose dose more effective than the lower dose. Although D-allulose is reported to reduce BW and liver fat in HFD-fed rats, the observed weight reduction with D-allulose plus dorsomorphin in rats requires a clear mechanistic justification. Corresponding mean ± SEM values at each time point, two-way ANOVA, and report both main effects and the treatment × time interaction provided in Supplementary Table S1.

**Figure 1:**
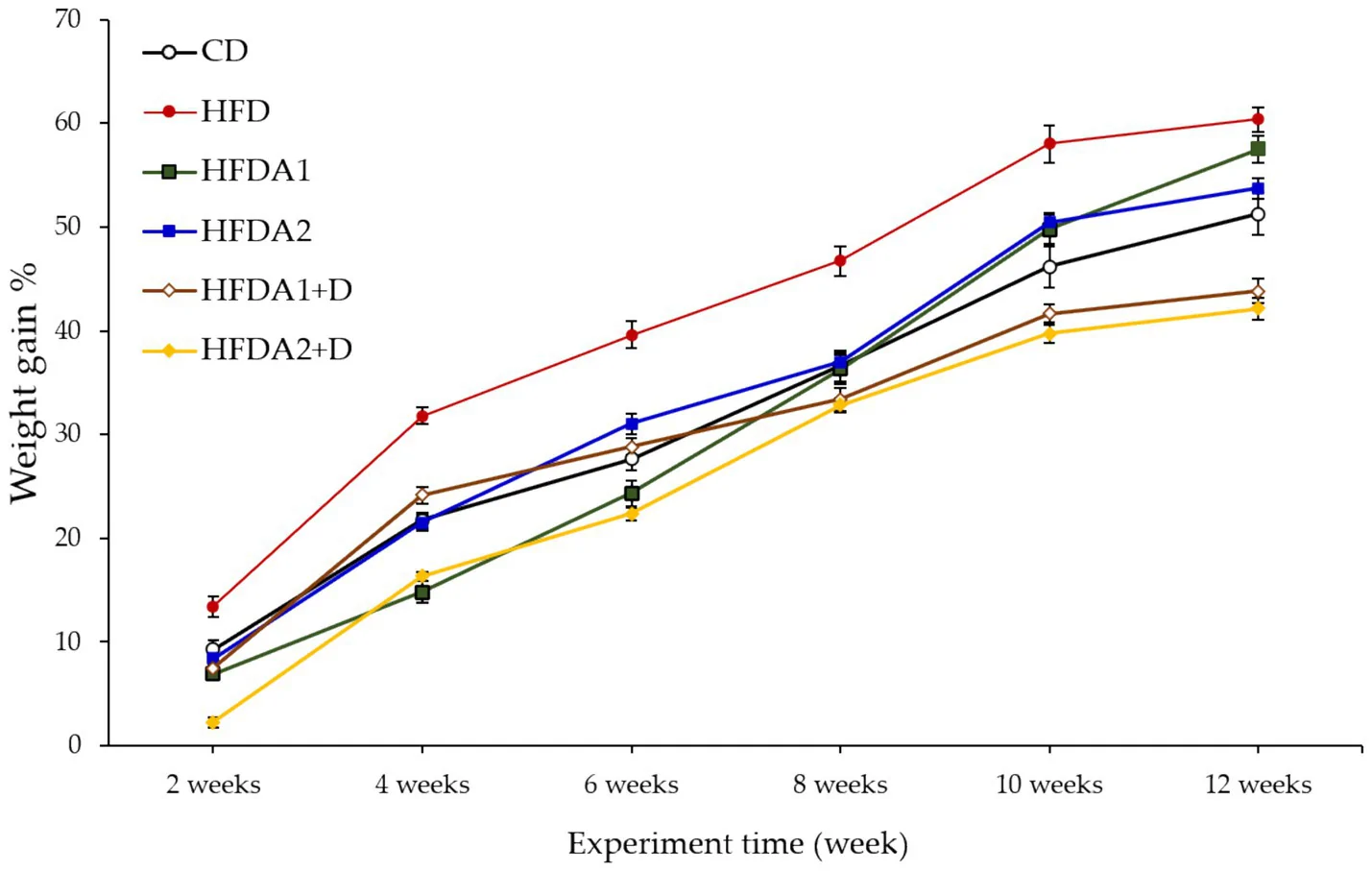
Effect of D-allulose alone or in combination with dorsomorphin on weight gain (%) in HFD-fed rats (mean±SEM), *n* = 8. CD: normal control group fed a standard chow diet; HFD: positive control group fed an HFD; HFDA1: HFD rats supplemented with oral D-allulose at 0.3 g kg^−1^ bw; HFDA2: HFD rats supplemented with oral D-allulose at 0.6 g kg^−1^ bw; HFDA1 + D: HFD rats supplemented with D-allulose at 0.3 g kg^−1^ bw plus dorsomorphin at 0.2 mg kg^−1^ bw orally; HFDA2 + D: HFD rats supplemented with D-allulose at 0.6 g kg^−1^ bw plus dorsomorphin at 0.2 mg kg^−1^ bw orally. See corresponding mean ± SEM values at each time point and two-way ANOVA reporting both main effects and the treatment × time interaction in Supplementary Table S1.

### Effect on serum lipid profile

Table 1 shows that the HFD group developed an atherogenic lipid profile characterized by higher TG, TCHOL, LDL-C, and VLDL-C levels than the control group. D-allulose supplementation produced dose-related reductions in these lipid parameters, and the lowest values among the treatment groups were generally observed in the high-dose combination group. The omnibus effects for TG, TCHOL, LDL-C, VLDL-C, and the HDL-C/TCHOL ratio remained significant after FDR correction (adjusted 𝑝≤0.009). In contrast, HDL-C concentrations did not differ significantly among the experimental groups (nominal 𝑝=0.460, adjusted 𝑝=0.506). Tukey’s pairwise comparisons are indicated by the superscript letters in Table 1.

### The ameliorative effects of D-allulose on the atherogenic index

Figure 2 indicates that the atherogenic index was markedly elevated in the HFD group compared with the normal control group, reflecting increased cardiovascular risk. In contrast, treatment with D-allulose alone or in combination with dorsomorphin significantly reduced this index toward control values. The lowest atherogenic index was observed in the high-dose combination group, suggesting that D-allulose, particularly at the higher dose, effectively attenuated the atherogenic burden induced by HFD.

**Figure 2:**
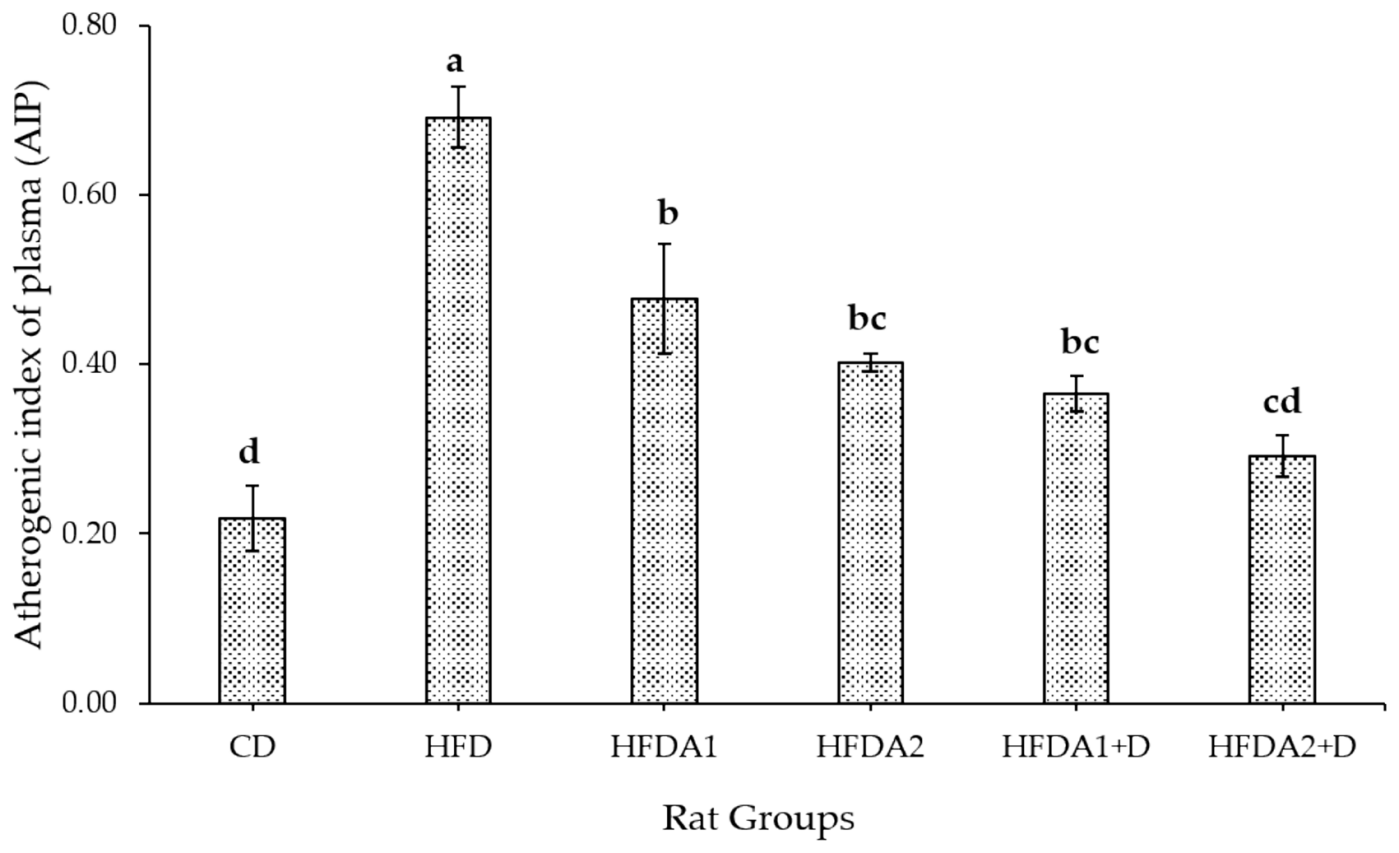
Effect of D-allulose alone or in combination with dorsomorphin on the atherogenic index of HFD-fed rats (mean±SEM). CD: normal control group fed a standard chow diet; HFD: positive control group fed an HFD; HFDA1: HFD rats supplemented with oral D-allulose at 0.3 g kg^−1^ bw; HFDA2: HFD rats supplemented with oral D-allulose at 0.6 g kg^−1^ bw; HFDA1 + D: HFD rats supplemented with D-allulose at 0.3 g kg^−1^ bw plus dorsomorphin at 0.2 mg kg^−1^ bw orally; HFDA2 + D: HFD rats supplemented with D-allulose at 0.6 g kg-1 bw orally plus dorsomorphin at 0.2 mg kg-1 bw. ^a,b,c,d^, Bars sharing the same letter do not differ significantly at *p* < 0.05 by one-way ANOVA followed by Tukey’s test.

### The ameliorative effects of D-allulose on the liver’s functions

As shown in Table 2, AST activity differed significantly among the experimental groups and remained significant after FDR correction (nominal 𝑝=0.001, adjusted 𝑝=0.002). Although ALT showed a borderline nominal difference among groups (𝑝=0.050), this endpoint did not remain significant after adjustment (adjusted *p* = 0.073) and should therefore be interpreted as exploratory. Total protein, albumin, and globulin concentrations did not differ significantly among groups before or after FDR correction. These findings suggest an improvement in selected indices of hepatic injury, although the evidence was stronger for AST than for ALT.

### The ameliorative effects of D-allulose on the kidneys’ functions

According to Table 3, neither the high-fat diet nor D-allulose, given alone or together with dorsomorphin, produced significant changes in serum creatinine, urea, or BUN (*p* > 0.05), and all groups shared the same superscript in the creatinine column, indicating preserved glomerular filtration across treatments. Urea and BUN showed only modest, non-significant trends toward lower values in the high-dose D-allulose group relative to HFD, suggesting that nitrogenous waste excretion remained within the physiological range and that the tested doses of D-allulose and dorsomorphin were not nephrotoxic, in line with reports that D-allulose alters renal metabolite profiles without impairing standard kidney function indices. Overall, the data suggest that nitrogenous waste excretion remained within the physiological range across all groups, with no clear evidence of HFD-induced azotemia or D-allulose-induced nephrotoxicity. Creatinine, urea, and BUN did not differ significantly among the experimental groups, either before or after FDR correction.

### The protective effects of D-allulose on the antioxidant biomarkers

As shown in Table 4, the HFD produced a marked oxidative stress pattern characterized by GSH depletion, MDA elevation, and reduced antioxidant enzyme activities. The omnibus differences for GSH, MDA, CAT, and SOD remained significant after FDR correction, with adjusted 𝑝-values of 0.002, 0.002, 0.029, and 0.002, respectively. D-allulose supplementation generally increased GSH levels and antioxidant enzyme activities while reducing MDA concentrations. The combination groups showed antioxidant outcomes comparable to or better than those observed with D-allulose alone. Thus, under the present experimental conditions, co-administration of dorsomorphin did not reverse the antioxidant protection conferred by D-allulose. Tukey’s pairwise comparisons are shown by the superscript letters in Table 4.

### The attenuative effects of D-allulose on pro-inflammatory cytokines TNF-Α, IL-6, and the insulin hormone

As shown in Table 5, IL-6, TNF-α, and insulin differed significantly among the experimental groups, and these differences remained significant after FDR correction, with adjusted 𝑝-values of 0.002, 0.002, and 0.006, respectively. HFD feeding was associated with higher cytokine and insulin concentrations, whereas D-allulose supplementation produced dose-related reductions in these endpoints. The combination groups generally exhibited the lowest levels of cytokines and insulin. FBG showed a borderline difference at the nominal level (𝑝=0.047); however, this finding did not retain significance after FDR correction (adjusted 𝑝=0.073). Therefore, the FBG result should be regarded as exploratory, although the treatment-group means showed a numerical pattern toward lower concentrations with D-allulose supplementation.

### The attenuative effects of D-allulose on liver structure

The microscopical examination of liver sections from the normal diet control group displayed normal hepatocytes with a central vesicular nucleus, normal central vein and blood sinusoids as well as normal portal triad (Figure 3A). In contrast, rats fed a high-fat diet exhibited congestion and dilation of the central vein, along with dilation and congestion of hepatic sinusoids with activated Kupffer cells (Figure 3B). Most hepatocytes showed marked degenerative changes and cytoplasmic vacuolation. Moreover, the majority of examined sections revealed hepatic micro and macro -vesicular steatosis (Figure 3B). Multifocal areas of coagulative necrosis exhibited hyperesinophilic cytoplasm with pyknotic nuclei were frequently seen (Figure 3C). Besides, focal aggregation of mononuclear leucocytic cellular infiltration were detected between necrotic hepatocytes (Figure 3D). Interestingly, the portal areas exhibited congestion and dilation of the portal vessels as well as hyperplasia of their lining epithelium with newly formed bile ductules accompanied by mononuclear inflammatory cells infiltrations, periductal fibrosis and periductal edema (Figure 3E). Meanwhile, the majority of the liver sections supplemented with oral D-allulose at 0.3 g kg^−1^ (HFDA1) showed small focal areas of coagulative necrosis with mild focal aggregation of mononuclear inflammatory cells (Figure 3F). In addition, supplemented with oral D-allulose at 0.6 g kg^−1^ BW (HFDA2), attenuation of these recorded histopathological lesions, where the hepatic necrosis and inflammation were markedly reduced. Only mild focal aggregation of mononuclear inflammatory cells in portal areas was recorded in a few examined sections, with the absence of newly formed bile ductules, periductal fibrosis, and periductal edema (Figure 3G).

**Figure 3:**
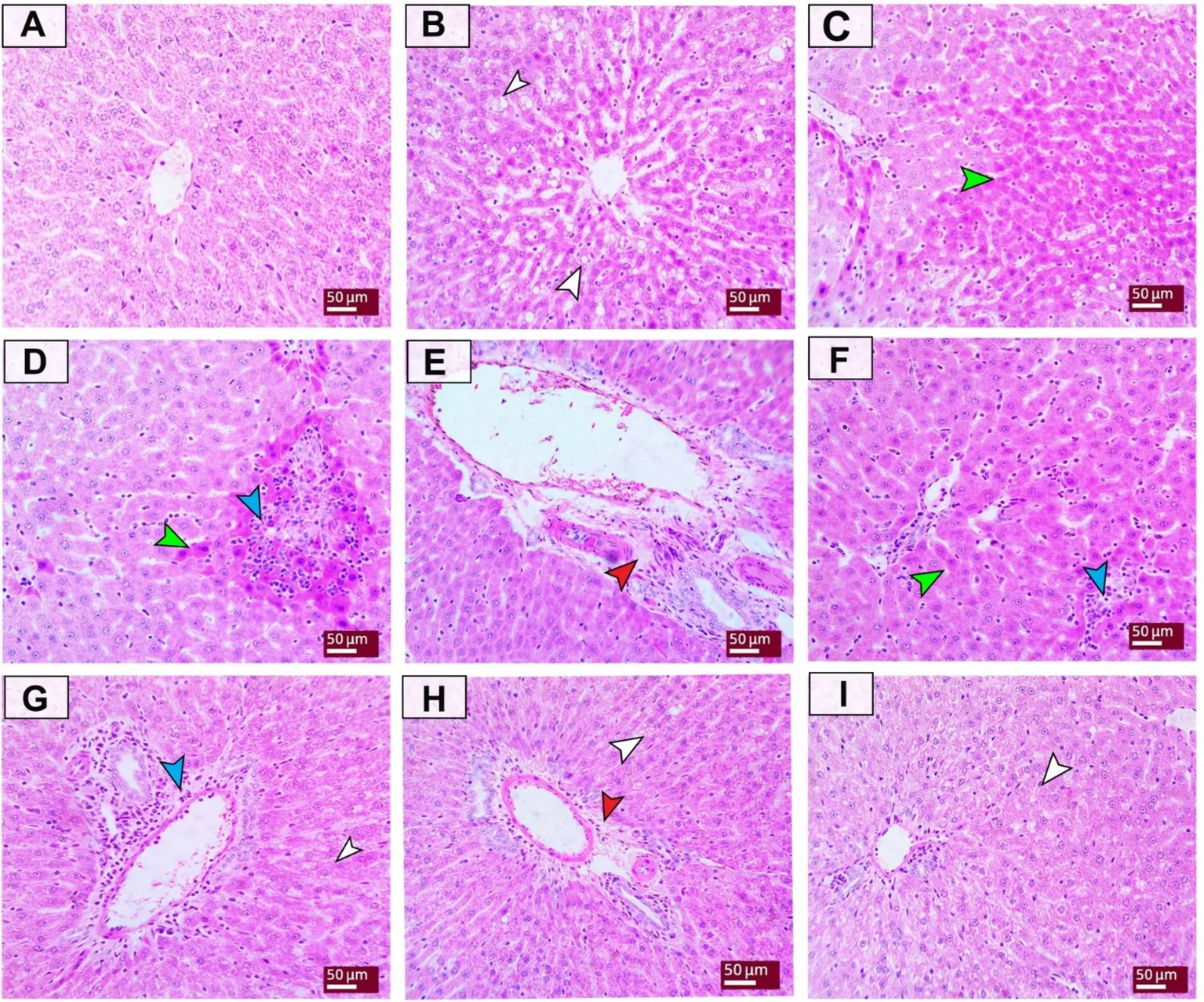
Representative photomicrographs of H&E-stained hepatic tissue. **(A)** control group, revealing normal histomorphology of the liver tissue. **(B-E)** Rats fed a high-fat diet showing **(B)** dilation of central vein and hepatic sinusoids with activated Kuffer cells along with severe micro and macro -vesicular steatosis, **(C)** diffuse coagulative hepatic necrosis represented with hypereosinophilic cytoplasm and pyknotic nucleus, **(D)** focal mono nuclear leucocytic cellular infiltration in between necrotic hepatocytes, **(E)** congestion and dilation of the portal vessels with newly formed bile ductules, mononuclear inflammatory cells infiltrations, periductal fibrosis and periductal edema. **(F)** supplementation with oral D-allulose at 0.3 g kg^−1^ (HFDA1) rats showing mild hepatic coagulative necrosis and fewer inflammatory cellular infiltration **(G)** rats orally administrated D-allulose at 0.6 g kg^−1^ BW (HFDA2) showing mild dilation of portal blood vessels and mild aggregation of mononuclear inflammatory cells in portal areas, **(H)** Supplementation with D-allulose (0.3 g kg^−1^, orally) together with 0.2 mg kg^−1^ BW dorsomorphin (HFDA1 + D) showing mild periductal lymphocytic infiltration and edema, **(I)** rats administrated D-allulose (0.6 g kg^−1^, orally) together with 0.2 mg kg^−1^ BW dorsomorphin (HFDA2 + D) showing mild hepatic vacuolar degeneration and micro vesicular steatosis. White arrow, micro and macro-vesicular steatosis; green arrow, pyknotic nuclei; blue arrow, mononuclear leucocytic cellular infiltration; red arrow, periductal edema (Bars = 50 μm).

Co-administration of D-allulose at 0.3 g kg^−1^ BW with dorsomorphin at 0.2 mg kg^−1^ BW (HFDA1D) markedly restored the normal hepatic architecture, with distinct hepatocytes, sinusoidal spaces, and central veins; only mild periductal inflammation and edema were occasionally observed (Figure 3H). Similarly, the HFDA2D group exhibited nearly normal histological architecture in most of the liver sections examined. Hepatocellular vacuolar degeneration, microvesicular steatosis, and coagulative necrosis were infrequently observed in this group (Figure 3I).

Histological lesions were semiquantitatively graded as shown in Table 6. HFD feeding caused marked hepatic injury characterized by vascular congestion, sinusoidal dilation, hepatocellular degeneration, steatosis, necroinflammation, and early fibrotic and ductular changes. D-allulose attenuated these histopathological alterations. Reassessment of the lesion-severity grades showed that co-administration of dorsomorphin did not reverse the histological protection. The HFDA1D and HFDA2D groups showed grades equal to or lower than those of HFDA2 for [3–5 of 9] evaluated lesion categories, consistent with the biochemical patterns observed in Tables 1, 4, 5. However, because no dorsomorphin-only group was included and AMPK activity was not directly measured, these histological findings are presented descriptively. They cannot establish whether the observed protection was AMPK-dependent or AMPK-independent.

**Table 6:** Lesion scores of histopathological changes in the liver of rats supplemented with HFD, D-allulose, and dorsomorphin.

Lesion	CD	HFD	HFDA1	HFDA2	HFDA1 + D	HFDA2 + D
Congestion/dilation of the central vein	−	+++	++	+	−	−
Sinusoidal dilation & Kupffer cell activation	−	+++	++	+	−	−
Hepatocellular vacuolar degeneration	−	+++	++	+	+	+
Micro/macrovesicular steatosis	−	+++	++	+	+	+
Coagulative necrosis	−	+++	++	+	−	−
Mononuclear leukocytic infiltration	−	+++	++	+	+	−
Portal vessel congestion/dilation	−	+++	++	+	+	+
Bile ductular hyperplasia	−	++	+	−	−	−
Periductal fibrosis	−	++	+	−	−	−
Periductal edema	−	++	+	+	+	−

CD, normal control group fed a standard chow diet; HFD, positive control group fed an HFD; HFDA1, HFD rats supplemented with oral D-allulose at 0.3 g kg^−1^ bw; HFDA2, HFD rats supplemented with oral D-allulose at 0.6 g kg^−1^ bw; HFDA1 + D, HFD rats supplemented with D-allulose at 0.3 g kg^−1^ bw orally plus dorsomorphin at 0.2 mg kg^−1^ bw; HFDA2 + D, HFD rats supplemented with D-allulose at 0.6 g kg-1 bw orally plus dorsomorphin at 0.2 mg kg-1 bw. Each lesion was graded as follows: (−): normal histology; (+): up to 1/3 of the section affected; (++): 1/3–2/3 affected; (+++): more than 2/3 affected. Lesion severity was graded as absent (−), mild (+), moderate (++), or severe (+++), which equal (0 for <5%), (1 for 5–33%), (2 for 33–66%), and (3 for >66%) according to Liang et al. (34).

## Discussion

Functional foods are increasingly recognized as important dietary strategies for preventing and managing obesity-related metabolic disorders because they can provide health benefits beyond basic nutrition. Among these, D-allulose has attracted attention as a rare sugar with low caloric value and promising bioactive properties, including effects on BW, lipid metabolism, oxidative stress, and inflammation. In the present study, the observed improvements in weight gain, lipid profile, liver function, status, and inflammatory markers support the concept that D-allulose acts as a functional food ingredient rather than a simple sugar substitute. These findings support the concept that D-allulose functions as a metabolically active sweetener rather than an inert sugar substitute (36–38).

The weight-gain trajectory in the D-allulose–treated groups is consistent with established anti-obesity effects of D-allulose in high-fat-diet (HFD) rodents, where it reduces BW gain, adiposity, and food efficiency (39, 40). These data therefore support the interpretation that D-allulose functions as an active metabolic modulator rather than a neutral sweetener (41). Lower weight gain likely reflects reduced energy intake, improved lipid oxidation and mitochondrial function, and attenuated adipose expansion, in line with mechanistic studies showing that D-allulose inhibits lipogenesis and is consistent with modulation of AMPK–SIRT1–PGC-1α–related pathways (23, 42). In the present experiment, parallel reductions in fat-tissue coefficient, improvements in lipid profile, and decreases in inflammatory cytokines and oxidative-stress markers support this integrated mechanism rather than an isolated effect on body mass (36, 37, 43). The stronger effect at the higher dose suggests dose-dependent efficacy, which is advantageous for translational development but may plateau at higher intakes, as reported for D-allulose and other rare-sugar or functional-food interventions (40, 42). The improved weight-gain profile in the D-allulose plus dorsomorphin groups should be interpreted cautiously, as dorsomorphin, although commonly used as an AMPK inhibitor, has multiple off-target actions, and the combined effect cannot be attributed solely to AMPK blockade (44, 45). In this context, D-allulose has been reported to improve MASLD through mechanisms that operate in parallel with or independently of the traditional AMPK/SIRT1 pathway (23), including regulation of miRNAs/macrophages and IFN-γ chemokine signaling, restoration of mitochondrial NADH homeostasis (18), activation of Nrf2 to inhibit NF-κB and ER stress (38), and hepatic metabolomic changes that enhance β-oxidation and bile acid synthesis (16). These effects establish D-allulose as a multi-target agent for hepatic steatosis and dyslipidemia (46). D-allulose suppresses weight gain, which is physiologically significant because weight trajectory predicts disorders such as dyslipidemia and insulin resistance; it also improves metabolic and inflammatory endpoints, offering broader protection than biomarkers alone (37, 47).

These findings confirm the successful induction of HFD-related dyslipidemia and indicate that D-allulose exerted an antihyperlipidemic effect rather than acting as an inert sweetener (23, 48, 49). Previous studies using rodent models, including HFD-fed animals, indicate that D-allulose may improve lipid metabolism and reduce adiposity through mechanisms that include suppression of hepatic lipogenesis, enhancement of fatty acid β-oxidation, and modulation of cholesterol handling; however, its effects on individual lipoprotein fractions appear to be model-dependent (41). In the present study, the reductions in triglycerides, LDL-C, and VLDL-C suggest decreased hepatic production of triglyceride-rich lipoproteins and/or improved clearance of atherogenic lipoproteins (16, 41, 50). These effects may involve PCSK9-related cholesterol pathways and hepatic lipoprotein uptake through scavenger receptor class B type 1 (49, 51). D-allulose may also influence HDL functionality; however, this remains unconfirmed, as HDL function was not directly assessed and HDL-C concentrations did not change significantly in the present study (23, 42, 44). Accordingly, these proposed mechanisms should be regarded as biologically plausible explanations rather than mechanisms demonstrated by the present experiment, and they require confirmation through direct molecular and functional analyses (18, 36, 37, 40, 42).

HFD induced marked hypertriglyceridemia and elevated LDL-C and VLDL-C, resulting in a higher atherogenic index. D-allulose significantly reduced these parameters in a dose-dependent manner, with the greatest reduction in the high-dose combination group. Thus, the present findings are consistent with evidence from high-fat-diet rodent models indicating that D-allulose may improve lipid metabolism and, under specific experimental conditions, reduce specific circulating lipoprotein fractions (23). An increase in the HDL-C/TC ratio generally indicates a more favorable lipid profile, even if absolute HDL-C changes are modest. MASLD, the hepatic manifestation of metabolic syndrome, is characterized by insulin resistance, which drives atherogenic dyslipidemia and typically presents with low HDL-C and elevated atherogenic lipid ratios, substantially increasing cardiovascular risk (13). In this context, the HFD-induced reduction in HDL-C/TCHOL and its normalization or surpassing of control values in the D-allulose-treated groups indicate a meaningful attenuation of MASLD-associated dyslipidemia and a shift toward a less atherogenic lipoprotein profile. Building on preclinical evidence that D-allulose improves adiposity, insulin sensitivity, and hepatic steatosis while modulating lipid handling, the observed increase in HDL-C/TCHOL, particularly at the highest dose, supports a mechanistic link between rare-sugar–mediated metabolic reprogramming and enhanced anti-atherogenic lipoprotein balance (52). Taken together, these findings position D-allulose-enriched functional foods as promising adjuncts to lifestyle and pharmacological MASLD management, offering partial sugar replacement with potential cardiometabolic benefit, especially in high-prevalence, sugar-rich dietary settings (52, 53). Because the atherogenic index of plasma (AIP) reflects mixed hyperlipidemia and cardiovascular risk, this suggests that D-allulose improves the overall lipid profile rather than affecting a single lipid fraction. The dorsomorphin effect should be interpreted with caution, as it is a non-selective signaling probe with off-target actions; therefore, the combination is best considered mechanistic rather than therapeutic (41, 52, 54).

HFD-induced liver injury was evidenced by elevated AST and bilirubin, with a trend toward higher ALT, consistent with MASLD-like hepatocellular damage reported in HFD rodent models. The unchanged ALP suggests predominantly hepatocellular rather than cholestatic injury (55, 56). D-allulose improved these abnormalities in a dose-dependent manner, normalizing AST and bilirubin and bringing ALT toward control values. This hepatoprotective effect is consistent with prior work showing that D-allulose reduces hepatic fat accumulation and modulates lipid metabolism, oxidative stress, and inflammatory signaling in obesity-related liver disease (16, 57, 58). The preserved total protein and albumin levels indicate that hepatic synthetic capacity was not impaired, supporting a favorable safety profile for D-allulose. The combination with dorsomorphin did not blunt protection, although dorsomorphin is a non-selective AMPK/BMP inhibitor, and its contribution should therefore be interpreted cautiously (44, 57, 59). Overall, the pattern suggests that D-allulose primarily acts on early mechanisms of MASLD, including lipid oxidation, lipogenesis, insulin sensitivity, and stress pathways. Compared with other HFD rodent models, the magnitude of benefit is notable, supporting D-allulose as a promising bioactive ingredient for obesity-related liver injury (60–62). In HFD models, D-allulose is a potent bioactive sweetener, often outperforming or comparing favorably to alternatives, though its physiological effects depend on the specific intervention (63). For example, compared to erythritol, D-allulose uniquely lowers postprandial blood glucose and enhances insulin sensitivity, while both improve gut microbiota profiles. D-allulose also shows superior anti-obesity effects via mitochondrial function (37, 64, 65). Compared with metformin, both reduce brain oxidative stress and improve learning, though metformin has an edge in addressing mitochondrial dysfunction (66, 67).

HFD did not significantly alter routine renal markers in this model, suggesting that any kidney injury remained subclinical and was not yet reflected in creatinine, urea, or BUN. D-allulose, alone or with dorsomorphin, did not impair renal function and may have offered subtle renoprotection, as the high-dose group showed numerically lower urea and BUN values. The absence of change in proteinuria-related markers also supports a kidney-safe profile for D-allulose. This is consistent with the renal safety profile of D-allulose observed in long-term animal studies (68, 69). Although minor renal tubular damage cannot be ruled out without urinary indicators, such as kidney injury molecule-1, KIM-1, and neutrophil gelatinase-associated lipocalin, NGAL (70). However, a dorsomorphin-only group is still needed to define the independent effect of the combination (68, 71–73).

HFD induced a clear state of oxidative stress, as evidenced by depleted GSH and elevated MDA levels, consistent with diet-related ROS overproduction and lipid peroxidation reported in HFD rodent models (74, 75). D-allulose reversed these changes in a dose-dependent manner, with the higher dose producing the strongest restoration of GSH and suppression of MDA, indicating robust antioxidant protection (23, 76, 77). In contrast to many reports showing parallel changes across multiple antioxidant enzymes, CAT activity in the present model was relatively stable, whereas SOD and GSH were more responsive (78–80). This pattern may reflect the differential sensitivity of these enzymes to chronic HFD-induced ROS or the specific timing and tissue compartment measured. It should therefore be interpreted cautiously as a somewhat atypical response rather than a fully resolved mechanistic finding. The concomitant decrease in GSH and SOD, alongside relative preservation of CAT activity, is consistent with chronic oxidative stress in which sustained ROS production depletes non-enzymatic antioxidants and impairs SOD, while catalase is not strongly upregulated as a compensatory response via redox-sensitive transcription factors. This pattern supports a shift toward glutathione- and SOD-dependent defense in HFD-induced oxidative stress, which D-allulose largely restored. The persistence of benefit in the dorsomorphin groups indicates that D-allulose’s antioxidant effect is resilient to pharmacologic perturbation of AMPK-related. However, dorsomorphin’s BMP-related off-target actions warrant cautious interpretation (81, 82). Overall, these findings support the use of D-allulose as a functional ingredient with redox-modulating potential in the context of HFD-induced metabolic stress (18, 36, 38, 77).

HFD induced a clear metabolic inflammatory state, as evidenced by marked increases in IL-6, TNF-α, and insulin, all of which are well-established features of obesity-related insulin resistance. D-allulose alleviated these abnormalities in a dose-dependent manner, and the combination with dorsomorphin generally produced the strongest suppression, suggesting pronounced anti-inflammatory and insulin-sensitizing effects (39, 83–85). The reduction in IL-6 and TNF-α is biologically important because these cytokines actively impair insulin signaling and promote metabolic deterioration (84). Their attenuation contributed to the improved insulin profile and may also help explain the concurrent benefits observed in lipid metabolism, liver function, and oxidative stress in the broader dataset (18, 43, 84). The marked increase in fasting insulin (~7-fold) with only a moderate rise in fasting glucose is consistent with early diet-induced insulin resistance, in which β-cells compensate by secreting more insulin to maintain near-normal glycemia (55, 85). The rise in insulin in the HFD group reflects compensatory hyperinsulinemia secondary to peripheral insulin resistance (85). In this context, the dose-dependent reduction in fasting insulin with D-allulose—most pronounced at 0.6 g kg^−1^ and in the combination groups—likely reflects improved whole-body insulin sensitivity mediated by reduced adiposity, attenuation of metaflammation (lower IL-6 and TNF-α), and improved hepatic and mitochondrial metabolic function rather than suppression of insulin secretion. Parallel improvements in weight gain, atherogenic lipids, and oxidative-stress markers support this integrated mechanism (18, 39, 43). Consistent with this, fasting blood glucose was only moderately elevated in HFD-fed rats compared with controls, despite the approximately 7-fold increase in insulin, indicating preserved but stressed β-cell function characteristic of early diet-induced insulin resistance. The partial normalization of both insulin and FBG in the D-allulose-treated groups, especially at the higher dose with or without dorsomorphin, therefore suggests an improvement in whole-body insulin sensitivity rather than a simple suppression of insulin secretion (85). Mechanistically, reductions in IL-6 and TNF-α are biologically relevant, as these cytokines contribute to insulin resistance and metabolic deterioration. Although AMPK-related signaling may contribute to these effects, the persistence of benefit in the dorsomorphin groups suggests that D-allulose may also act through AMPK-independent or broader metabolic pathways (23, 59). Although dorsomorphin is an inhibitor, the persistence of benefit in the combination groups suggests that D-allulose-activated AMPK acts through broader metabolic and immune pathways. Because dorsomorphin is a non-selective inhibitor with known off-target effects, it should be treated as a mechanistic probe rather than proof of AMPK blockade alone (81–83).

The histopathological findings confirmed that HFD induced clear MASLD-like liver injury, whereas D-allulose dose-dependently reduced steatosis, necrosis, inflammation, and early fibrosis, indicating a shift from steatohepatitis-like damage toward improved hepatic histology (86, 87). Steatosis reflects hepatocellular lipotoxic stress, and the associated vacuolar degeneration, necrosis, and Kupffer-cell activation indicate progression to an active necroinflammatory phase (88). Portal congestion, ductular hyperplasia, edema, and periductal fibrosis indicate a more advanced fibro-inflammatory MASLD phenotype than simple lobular steatosis (89, 90). The histopathological findings were consistent with the biochemical results. Contrary to our original interpretation, co-administration of dorsomorphin did not reverse the histological protection conferred by D-allulose. The HFDA1D and HFDA2D groups showed lesion-severity grades equal to or lower than those of HFDA2 in [3–5 of 8] evaluated categories. This pattern parallels the lipid, antioxidant, and inflammatory findings in Tables 1, 4, 5, in which the combination groups generally matched or outperformed D-allulose alone. Therefore, the present results do not support a simple interpretation in which dorsomorphin-mediated AMPK blockade weakened the protective effects of D-allulose. Nevertheless, these findings do not demonstrate that AMPK is uninvolved, because dorsomorphin is a non-selective pharmacological inhibitor, systemic AMPK inhibition was not directly confirmed, and a dorsomorphin-only group was not included. Accordingly, the contribution of AMPK remains unresolved and requires confirmation using direct measurements of AMPK phosphorylation and more selective pharmacological or genetic approaches.

D-allulose clearly improved liver architecture, with a stronger effect at 0.6 g kg^−1^ than at 0.3 g kg^−1^. The lower dose reduced focal necrosis and inflammation, whereas the higher dose largely preserved hepatic structure and removed most portal inflammatory and fibrotic changes. This dose–response pattern supports a real hepatoprotective effect and is consistent with biochemical improvements in lipid profiles, liver enzymes, oxidative stress, and inflammatory markers. The near-normal histology in the high-dose D-allulose plus dorsomorphin group is especially noteworthy. Despite AMPK inhibition, the liver architecture remained largely preserved, suggesting that D-allulose may act through multiple overlapping pathways rather than a single signaling pathway. Overall, these histological data show that D-allulose not only improved serum markers but also directly protected hepatic tissue from HFD-induced structural injury.

Collectively, the lesion scores indicate that D-allulose substantially mitigates HFD-induced steatosis, inflammation, and fibrotic remodeling. Contrary to a simple AMPK-dependence model, the dorsomorphin combination groups did not show worse histology than D-allulose alone; scores were generally equal to or lower, mirroring the biochemical pattern reported in Tables 1, 4, 5. Because no AMPK-specific assay was performed and no dorsomorphin-only arm was included, these histological data cannot establish a causal link to hepatic AMPK activity; instead, they are more consistent with AMPK-independent or off-target dorsomorphin contributions, and should be interpreted alongside previous reports that D-allulose can act via AMPK-SIRT1–PGC-1α signaling as well as parallel, AMPK-independent pathways in high-fat models (16, 17). Furthermore, dorsomorphin is a well-characterized AMPK and BMP type I receptor inhibitor used to dissect AMPK-dependent hepatoprotective effects, and similar HFD-induced histopathological patterns—central vein congestion, sinusoidal dilation, steatosis, and portal fibrosis—have been extensively documented in rat MASLD (formerly NAFLD) models, reinforcing the translational relevance of the present data (91).

From a translational perspective, the oral D-allulose doses used in this MASLD model (0.3 and 0.6 g/kg in rats) correspond to approximate human-equivalent doses of about 0.05 and 0.10 g/kg, respectively, based on standard body-surface-area conversion (rat Km = 6, human Km = 37), i.e., roughly 3–7 g day^−1^ for a 60–70 kg adult. Short-term human studies have generally reported that D-allulose is well tolerated at comparable or lower daily intakes, with modest improvements in postprandial glycemia and insulin, and mainly mild, dose-dependent gastrointestinal symptoms at higher doses (92). In line with these data, our findings support D-allulose as a promising functional ingredient targeting dyslipidemia, oxidative stress, and low-grade inflammation; however, well-designed clinical trials at realistic dietary doses and over longer durations are still required before firm recommendations can be made for human MASLD prevention or management.

Because the present study evaluated a broad panel of biochemical endpoints, we conducted a retrospective Benjamini–Hochberg sensitivity analysis across the 22 omnibus tests reported in Tables 1–5. The principal findings for atherogenic lipids, AST, antioxidant status, inflammatory cytokines, and insulin remained significant after FDR correction. CAT also retained significance, although its adjusted 𝑝-value was closer to the 0.05 threshold. In contrast, the nominal findings for ALT and FBG did not remain significant after correction and should therefore be regarded as exploratory. These results support interpreting the study primarily by the consistent pattern across related metabolic and histological endpoints, rather than relying on isolated borderline findings.

This study has several limitations. The proposed mechanisms were not directly evaluated because hepatic AMPK activation and downstream molecular targets related to lipid metabolism, oxidative stress, and inflammation were not measured. Moreover, food intake, feed efficiency, fecal lipid excretion, and direct indices of insulin sensitivity were not assessed. The oral pharmacokinetics and systemic inhibitory effects of dorsomorphin were also uncertain, and the absence of a dorsomorphin-only group prevented separation of its independent effects. Given its non-selective inhibition of AMPK/BMP and off-target effects, dorsomorphin should be considered an exploratory pharmacological probe, and the combination results do not establish either AMPK-dependent or AMPK-independent action. Finally, multiplicity control was not prespecified. A retrospective Benjamini–Hochberg sensitivity analysis was therefore applied to the 22 rounded omnibus 𝑝-values reported in Tables 1–5; consequently, the adjusted values are approximate, and the nominal ALT and FBG findings that did not remain significant should be considered exploratory. Future studies should incorporate prespecified primary outcomes, exact 𝑝-values, direct molecular and insulin-sensitivity measurements, food intake and fecal lipid assessments, more selective or genetic approaches, and a dorsomorphin-only group. Future studies should therefore incorporate comprehensive molecular analyses, more selective inhibitors, a dorsomorphin-only treatment arm, appropriate genetic approaches, and direct insulin-sensitivity testing to better define the underlying mechanisms and strengthen causal inference.

## Conclusion

D-allulose was associated with improvements in several lipid, antioxidant, inflammatory, endocrine, and histopathological outcomes in HFD-fed rats. The principal effects on atherogenic lipids, oxidative-stress biomarkers, inflammatory cytokines, and insulin remained significant in a retrospective FDR sensitivity analysis. In contrast, the findings for ALT and FBG should be considered exploratory. Co-administration of dorsomorphin did not reverse the biochemical or histological protection associated with D-allulose. Nevertheless, the absence of a dorsomorphin-only group, the lack of direct verification of AMPK inhibition, and the retrospective use of multiplicity correction limit the mechanistic and confirmatory interpretation of the results. Additional studies with prespecified primary endpoints, direct molecular measurements, and prospectively defined multiplicity-control procedures are warranted. Future work should use direct molecular analyses, more selective inhibitors, or genetic approaches, gut microbiota profiling, and longer-term translational studies. Overall, the study strengthens the evidence that D-allulose is a promising bioactive sweetener for addressing obesity-associated metabolic stress.

## Data Availability

The original contributions presented in the study are included in the article/Supplementary material, further inquiries can be directed to the corresponding author.
