# Supplementary material for: D-allulose attenuates dyslipidemia, oxidative stress, and inflammation in high-fat-diet-induced metabolic dysfunction-associated steatotic liver disease in rats
Source: Front Nutr. 2026 Oct 14;13:1982241. doi: 10.3389/fnut.2026.1982241 (PMC13713509; doi:10.3389/fnut.2026.1982241)
Supplement: Supplementary file 1 [file Data_Sheet_1.pdf]

Table S1. Effect of D-allulose alone or in combination with dorsomorphin on weight gain (%) in HFD-fed rats (mean±SE), *n*=8.

	2 weeks	4 weeks	6 weeks	8 weeks	10 weeks	12 weeks
**CD**	9.29<br>±0.84	21.74<br>±0.76	27.62<br>±1.10	36.60<br>±1.53	46.14<br>±1.94	51.26<br>±2.04
**HFD**	13.40<br>±0.99	31.79<br>±0.80	39.63<br>±1.27	46.73<br>±1.40	58.02<br>±1.79	60.37<br>±1.16
**HFDA1**	6.95<br>±0.65	14.79<br>±1.07	24.34<br>±1.23	36.28<br>±1.46	49.75<br>±1.43	57.46<br>±1.30
**HFDA2**	8.40<br>±0.56	21.50<br>±0.74	31.03<br>±0.99	36.99<br>±0.87	50.47<br>±0.93	53.73<br>±1.03
**HFDA1+D**	7.50<br>±0.78	24.15<br>±0.83	28.83<br>±0.81	33.37<br>±1.07	41.67<br>±0.88	43.82<br>±1.19
**HFDA2+D**	2.28<br>±0.51	16.34<br>±0.44	22.36<br>±0.63	32.87<br>±0.68	39.71<br>±0.89	42.12<br>±1.07

CD: normal control group fed a standard chow diet; HFD: positive control group fed an HFD; HFDA1: HFD rats supplemented with oral D-allulose at 0.3 g kg^{-1} bw; HFDA2: HFD rats supplemented with oral D-allulose at 0.6 g kg^{-1} bw; HFDA1+D: HFD rats supplemented with D-allulose at 0.3 g kg^{-1} bw plus dorsomorphin at 0.2 mg kg^{-1} bw orally; HFDA2+D: HFD rats supplemented with D-allulose at 0.6 g kg^{-1} bw plus dorsomorphin at 0.2 mg kg^{-1} bw orally.

Table S2. Formulation of normal and high-fat-diets (HFD)

Caloric data	Control Diet	High Fat Diet
**Protein**	20% kcal	20% kcal
**Fat**	10% kcal	60% kcal
**Carbohydrate**	70% kcal	20% kcal
**Energy Density**	3.82 kcal/g	5.21 kcal/g
**Ingredient**		
**Casein**	200.00 g	200.00 g
**Methionine**	0.00 g	0.00 g
**L-Cystine,**	3.00 g	3.00 g
**Starch, Corn**	506.20 g	0.00 g
**Lodex 10**	125.00 g	125.00 g
**Sucrose**	72.80 g	72.80 g
**Fiber**	50.00 g	50.00 g
**Lard**	20.00 g	245.00 g
**Soybean Oil, USP**	25.00 g	25.00 g
**Butter, Anhydrous**	0.00 g	0.00 g
**Corn Oil**	0.00 g	0.00 g
**Mineral (S10001A)**	50.00 g	50.00 g
**Calcium Phosphate**	0.00 g	0.00 g
**Calcium Carbonate**	0.00 g	0.00 g
**Choline Bitartrate**	2.00 g	2.00 g
**Vitamin(V10001C)**	1.00 g	1.00 g
**Ethoxyquin**	0.00 g	0.00 g
**Cholesterol, NF**	0.00 g	0.00 g
***Cholesterol from lard**	0.02 g	0.23 g
**#Cholesterol from butter**	0.00 g	0.00 g

* Typical analysis of cholesterol in lard = 0.95 mg/gram

#Anhydrous milk fat typically contains approximately 0.3% cholesterol.
